# Amyloid Precursor Protein Expression Level Influences Mitochondrial Functioning and Differentially Impacts Protein Expression of Mitochondrial Quality Control Markers

**DOI:** 10.1002/alz.095588

**Published:** 2025-01-09

**Authors:** Joseph Pleen, Ryan Goodson

**Affiliations:** ^1^ University of Kansas Alzheimer’s Disease Research Center, Fairway, KS USA

## Abstract

**Background:**

Recent work suggests that amyloid precursor protein (APP) may be involved in regulating mitochondrial quality control mechanisms. Impaired mitophagy, leading to the accumulation of damaged mitochondria are features of Alzheimer’s disease (AD). Conversely, enhancing mitophagy may reduce AD neuropathological change and improve cognitive function. Understanding the interplay between APP processing, mitochondrial dysfunction, and mitophagy holds promise for developing novel therapeutic strategies for AD. By targeting these pathways, we may be able to reduce AD pathology, improve mitochondrial function, and ultimately slow or prevent the progression of AD.

**Method:**

Utilized CRISPR/Cas9 gene editing to knock out the APP gene in SH‐SY5Y cells. We also utilized small interfering RNA (siRNA) to transiently reduce the expression of APP in SH‐SY5Y. This was compared to controls and another model of mitochondrial dysfunction the SH‐SY5Y Rho0 cells which are depleted of mitochondrial DNA. Western blots and capillary electrophoresis were used to measure APP levels and protein markers of mitophagy. The Mitochondrial Stress Test (MST) assay was performed on a Seahorse XF analyzer to assess the impact of APP levels on the functional capacity of mitochondria.

**Result:**

Protein levels of SQSTM1/p62 and NDP52 were significantly reduced with APP siRNA knock down (APP KD) but elevated in APP knockout (APP KO) SH‐SY5Y cells compared to control on both capillary electrophoresis and western blots. Rho0 cells also had significantly elevated SQSTM1/p62 protein, but not to the degree of the APP KO cells. Functionally the APP KO cells had reduced oxygen consumption rate and maximal respiration, almost to the level of Rho0 cells. The APP KD cells showed a trend that did not reach significance.

**Conclusion:**

Our data suggests that APP serves a vital role in normal mitochondrial functioning or mitochondrial quality control as evidenced by the reduced oxygen consumption rate and maximal respiration in APP KO cells. Additionally, this role may be mediated through impaired autophagy/mitophagy as accumulation of SQSTM1/p62 is a known marker of impaired mitophagy. NDP52 can also recognize and bind to ubiquitinated phosphorylated Tau (pTau), potentially targeting it for autophagic degradation, which may be a link between the two major pathological hallmarks of AD (Aβ and pTau).

